# Assessment and Management of Postoperative Pain among Nurses at a Resource-Constraint Teaching Hospital in Ghana

**DOI:** 10.1155/2019/9091467

**Published:** 2019-07-18

**Authors:** Faisal Mahama, Jerry P. K. Ninnoni

**Affiliations:** ^1^Department of Nursing, Holy Family Nursing and Midwifery Training College, Ghana; ^2^Department of Mental Health, School of Nursing and Midwifery, University of Cape Coast, Ghana

## Abstract

**Background:**

Postoperative pain remains one of the greatest concerns for patients following surgical procedures. Nurses play an essential role in postoperative pain assessment and management, especially within the first few days after surgery.

**Objective:**

The study investigated how nurses in a resource-constraint hospital in Ghana assessed and managed postoperative pain.

**Methods:**

This was an explorative qualitative study involving 12 registered nurses practising in the largest referral hospital in Ghana. Data was gathered using a semistructured interview guide. Demographic characteristics of participants were summarized using descriptive statistics. Data were analysed using Kvale's three phases for analysing qualitative data. First, the entire text was read again to identify meaning units which were then condensed. Second, the condensed texts were read again and interpreted. Finally, the condensed data containing similar meaning were coded and then sorted into subthemes.

**Results:**

It was found that some nurses have never used any pain assessment tool due to lack of standard tool for assessing postoperative pain. The majority of nurses reported that managing pain by using medication was the norm especially in the first 24 hours after surgery.

**Conclusion:**

Although participants may have some knowledge of assessing and managing postoperative pain, this knowledge was not largely used to manage postoperative pain effectively, partly because of resource constraints. Therefore, there is the need for adequate training and with provision of resources, it is imperative that the use of standardized pain assessment scales could help in the proper assessment and management of postoperative pain in this setting.

## 1. Introduction

Pain is a complex phenomenon and has been variously defined by clinicians, researchers, and patients [1]. The International Association for the Study of Pain defined pain as an unpleasant sensory and emotional feeling or experience associated with actual or potential tissue damage [2]. Alternatively, McCaffery and colleague advocate that pain is the patient's reported experience [3]. Postoperative pain is perceived as pain of recent onset and probable limited duration [4]. It is claimed that 50% of patients experience pain within 24 hours following surgery and gradually decreases within a few days after the surgery [5, 6].

Effective pain assessment is attributed to be the foundation for effective pain management and must be routinely undertaken for all postoperative procedures [4, 7]. Patients' responses to pain are subjective and dependent on the individual and thus should be evaluated on an individual basis. However, a study result indicated that over 57% of nurses had insufficient knowledge regarding tools that may be employed for pain assessment and measurement and 12% of the health care providers had never used any tools to assess pain [8]. Aziato, Dedey, Marfo, Asamani, and Clegg-Lamptey [9] conducted a mixed method study in Ghana to validate the use of three pain assessment scales among adult postoperative patients. They observed that using a valid tool for pain assessment gives the clinician an objective criterion for pain management.

Poorly managed pain can have a negative impact on physical and psychological consequences for patients and their carers [10]. Therefore, nurses have a professional and ethical responsibility to ensure effective pain relief for their patients. Optimal pain relief is dependent on nurses' knowledge and understanding of pain including systematic and consistent assessment and regular observation and documentation of pain (Francis & Fitzpatrick, 2013). Pain management is considered by patients as a right and an expectation [11]. However, postoperative pain remains one of the greatest concerns for patients following surgical procedures. Numerous studies ([8, 12]; Francis & Fitzpatrick, 2013) have demonstrated the inadequacy of pain management across all clinical settings and this could be higher in settings such as Ghana where resources are limited. Aziato and Adejumo [13] conducted a qualitative study with a clinical ethnographic approach that was aimed at exploring the perceptions and responses of Ghanaian surgical nurses regarding patients' postoperative pain. The results indicated that nurses had subjective views regarding postoperative pain and responded to patients' pain by administering analgesics and nonpharmacologic measures.

Postoperative pain is a major health care problem [14, 15]. Inadequately managed postoperative pain is prevalent worldwide which adversely affects patients' experience and outcome [16, 17]. In Sub-Sahara Africa, pain has been studied largely in relation to HIV/AIDS and cancer [18–20]. Although postoperative pain poses a similar burden, limited studies investigated postoperative pain in Ghana [9, 13]. Thus, a gap exists in the area of research regarding nurses' understanding, assessment, and management of postoperative pain. This study explored nurses' understanding, assessment, and management of postoperative pain in Ghana.

## 2. Materials and Methods

### 2.1. Ethics

Prior to the commencement of data collection, a formal request for ethical approval was made to the necessary institutions and departments. Firstly, permission to proceed with the study was granted from the School of Nursing and Midwifery and ethical approval obtained from University of Cape Coast Institutional Review Board. The study was also approved by Institutional Review Board of Korle-Bu Teaching Hospital.

### 2.2. Design

An explorative qualitative design was chosen for this study. The study explored the knowledge and practices of nurses on postoperative pain assessment and management in patients. This study aimed to understand the knowledge and practices of postoperative pain assessment and management provided by nurses working in the surgical intensive care unit.

### 2.3. Setting

The study was carried out at Korle-Bu Teaching Hospital (KBTH), which is situated in the southern part of Ghana. The KBTH is the premier teaching hospital in Ghana and the leading national referral hospital in the country. It has a bed capacity of approximately 2,000. It is currently the third largest Hospital in Africa. However, resources and logistic to facilitate nursing care remain a challenge.

### 2.4. Population and Sampling Technique

The population of nurses at hospital was 1,324 [21]. Purposive sampling technique was employed to recruit participants for the study. In purposive sampling strategy, researchers choose the cases to be included in the sample on the basis of a judgement of their typicality or possession of the particular characteristics being sought after [22]. The researcher focussed on sampling participants with various experiences who worked at the Surgical Intensive Care Unit of the hospital and are

1. registered with the Ghana Nurses and Midwives Council,
2. employed by Korle-Bu Teaching Hospital,
3. able to give a written consent to participate in the study,
4. employed at the hospital for at least one year.

Twelve (12) registered nurses were interviewed to reach saturation. Data saturation is reached when there is enough information and no new information emerging or when further coding is no longer feasible [23, 24].

### 2.5. Data Collection Instrument and Process

To explore nurses' knowledge and practices related to assessment and management of postoperative pain, data were collected by tape-recorded semistructured interviews with the participants consent. The semistructured interview guide comprised both open and closed ended questions. Questions posed were placed under three main sections. These included

1. Section A: nurses' understanding of postoperative pain;
2. Section B: nurses' assessment of postoperative pain;
3. Section C: nurses' management of postoperative pain.

The researcher conducted all the interviews at the hospital, as agreed with the participants. Each interview lasted for approximately one hour. Prior to the commencement of the interviews, the researcher explained the purpose of the study and confidentiality ensured. The interviews begun after written informed consent from the interviewee was sought. For most part of the interview, open ended questions were asked. The researcher maintained a nonjudgmental approach and observed both verbal and nonverbal messages during the interviews.

### 2.6. Data Analysis

Analysis of the data took place as soon as each data set was collected. All interviews were taped and transcribed verbatim. Analysis followed Kvale's [25] three phases for analysing qualitative data (Table 1). In the first phase, each transcript was read several times to gain a fuller understanding of the content. Following this, the whole text was read again and the meaning units were identified and condensed. In the next phase, texts were reread, analysed, and classified within the nursing context and guided by the purpose of the study. In the third phase, condensed data that contained the same meaning were coded and then sorted as subthemes. Those subthemes that were related to each other were grouped into three distinctive themes.

### 2.7. Rigor

Lincoln and Guba [26] forwarded four principles regarding the trustworthiness of qualitative research. These include: credibility, transferability, dependability and confirmability. The credibility of this study was ensured by basing the interview guide on one created from a pilot interview conducted with a similar group. The interview guide was designed to reflect the aim and objectives of the study. To achieve credibility, the data, coding, and the emerging themes were discussed with supervisors regularly throughout the analysis. Transferability was achieved through detailed description of the study design, including participants' selection, method of data analysis, and the context of the study to inform any possible replication of this study. With respect to confirmability, the researchers are nurse educators and a public health practitioner. The main supervisor is a mental health nurse specialist and the student is a nurse educator. In qualitative studies, it is claimed that the knower and known are interactive and inseparable [26]. Having been nurses at early stages of our careers, the researchers are aware of their own biases and how this can influence the data. The only way of ensuring the findings are credible is by suspending or bracketing our own beliefs and values and allowing the data to be emergent. Also to ensure confirmability, all the filed notes, audios, and transcripts were reviewed by the supervisory team for agreements. The study is detailed enough with clear audit trail for any external person to be able to follow the reasoning and the conclusions.

## 3. Results

### 3.1. Demographic Characteristics of Participants

Study demographics involved were three males and nine females with their ages ranging from 20 years to above 40 years (Table 2). They had clinical nursing experience of 1 to over 10 years. Participants in the study included staff nurses (2), senior staff nurse (1), nursing officers (4), senior nursing officers (4), and principal nursing officer (1). Eight of the nurses had training in critical care nursing whilst three of them had some form of workshop on pain.

Analysis of data resulted in three themes which address nurses' knowledge and practices related to assessment and management of postoperative pain in the hospital. These themes are as follows: 1. nurses' understanding of pain and postoperative pain; 2. nurses' assessment of postoperative pain; and 3. nurses' management of postoperative pain.

### 3.2. Theme One: Nurses' Understanding of Pain and Postoperative Pain

#### 3.2.1. Defining Pain

The subjective nature of pain was commonly reported by participants. Pain was viewed as unique to each patient and could be best described by the person experiencing it.

Errrrm relatively, I think pain is what the person, the patient says it is. I won't have a particular definition for it. If a patient mentions that he is in pain it means that he is in pain irrespective of what the health worker thinks or knows. (N1)

With my little knowledge of pain, I believe pain is basically determined by the patient not the health worker because the patient is the person experiencing the kind of stimuli and how the body is reacting to it and I believe pain is individualized, different people reacting, different stimuli and in a different way. (N8)

#### 3.2.2. Defining Postoperative Pain

Postoperative pain is an expectation of both nurses and patients. The participants believed that patients go into surgery anticipating that when they wake up from anaesthesia, they will experience pain. However, participants empathized with the patients having postoperative pain and acknowledged the need for relief of patient's pain.

Post-operative pain is a pain that a patient experiences after having gone or undergone a surgical operation. (N9)

Postoperative pain is a pain that one gets due to surgical intervention after the patient has been operated upon and is in the recovery ward or is in the ICU or has come back to the ward. (N11)

### 3.3. Theme Two: Nurses' Assessment of Postoperative Pain

This theme describes measures that are undertaken by nurses to ensure optimal pain assessment. Participants reported verbalisation, vital signs, and body language as some of the ways of identifying and assessing pain. It appears that patient's facial expression was often one of the first indicators that the patient is experiencing postoperative pain.

Well, errrrm, post-operative pain it could be verbalized, sometimes it becomes physiologic, through their vital signs you could see raised blood pressure, and then the heart rate will also rise, respiration will also increase and then you would know that patient is uncomfortable. Sometimes it becomes obvious errrrm by their appearance, through the facial expression, that patient is uncomfortable you ask and then they are still in pain. (N3)

When asked about pain assessment scales some of the nurses stated that they have never used any pain assessment tool before and that the unit has no standard tool for assessing postoperative pain.

Not always. Sometimes you ask the patient, “are you in pain?” and the patient says “yes or no”, that's all. (N10)

We don't have a standard tool. Whatever you use is based on your own discretion; that which will help you to help the patient. (N7)

### 3.4. Theme Three: Nurses' Management of Postoperative Pain

#### 3.4.1. Pharmacological Treatment of Postoperative Pain

The majority of nurses reported that managing pain by using medication prescribed by the physician was what they always do for the patients, especially in the first 24 hours after surgery.

You know mostly you have to use your discretion. A doctor may write or prescribe a medication maybe either the dose is too high. You the nurse you have to be smart and then give the correct dose or give the dose that is not more than what has been ordered. (N9)

Management with medication, normally we go by orders from either the anaesthetist or the surgeon. (N3)

Participants also reported that drugs commonly used after surgery include analgesics such as opioids analgesics, nonopioid analgesics, multimodal analgesics, and adjuvants. Intravenous paracetamol was noted to be commonly used in the treatment of postoperative pain because of its quick onset of action and can considerably provide more sparing opioid effect.

We can give morphine, we can give Midazolam, we can give pethidine, we can give IV paracetamol, we can give suppository paracetamol or diclofenac. (N4)

We have pethidine IM or IV sometimes we combine it, we have morphine, we have fentanyl, we have IV paracetamol, we have suppository paracetamol, we have diclofenac IM or suppository diclofenac and then we have tablets too. (N6)

#### 3.4.2. Nonpharmacological Management of Postoperative Pain

Another facet to nursing management of postoperative pain is the incorporation of nonpharmacological measures in managing postoperative pain. Diversional therapy, relaxation, and music therapy are some methods employed by nurses to manage postoperative pain.

Sometimes with ICU patients, not particularly for the pain but with ICU patients who are recovering, who have been on the ward for a very long time we ask their families the kind of music they are interested in, sometimes we report to them to bring their radio sets and then we try to play them some music… (N1)

With regard to the use of the nursing process/care plan in the management of postoperative pain, most of the participants did not make use of it when providing care. Some participants indicated that the use of the nursing process/care plan was carried out abstractly.

This place we don't normally go according to the nursing care plan or nursing process. That's what initially I told you that we do, we care for the patient, if you say you are going according to the nursing process or nursing care plan, you can, you'll follow a steps but we do according to priority. (N4)

We don't have a structured this thing but it's in our head; you do your, you plan, you assess, you intervene, you analyse and you see whether you are able to relieve the pain or not and if not then you find alternate ways or any other way of relieving the pain. (N6)

## 4. Discussion

The findings revealed that nurses in this premier Ghanaian teaching hospital shared a common view on the definition of pain as reported in the literature. All definitions reflect the subjective nature of pain which is unique to each patient. They considered pain to be an occurrence that can only be best described by the one experiencing it. These participants' description of pain is aligned to McCaffery [27] who unambiguously refers to pain as whatever the person says it is and existing whenever the experiencing person says it exists. This underpins the individual's unique concept of pain and thus informs holistic care. It is worth noting that participants made no emphasis on the universally acknowledged and accepted definition of pain which is “an unpleasant sensory and emotional experience associated with actual or potential tissue damage or described in terms of such damage [6].”

Both nurses and patients expect some form of pain after surgery. The participants believed that patients go into surgery anticipating that when they wake up from anaesthesia, they will experience pain. Tissue damage is one of the main sources for acute pain and the sensation of pain warns the body that it has been injured [28]. Postoperative pain is a prevalent type of acute pain, which is referred to as a pain of recent onset and with probable limited duration [4]. It can therefore be deduced from literature that acute pain following surgery is a predictable, physiological response to tissue damage as stipulated by participants.

The assessment of pain is an essential nursing activity in postoperative pain management and involves communication between the patient and the nurse. The pain assessment methods available to the nurse include observation, physiological responses, self-report from the patient using pain scales, location, and intensity of the pain and assessing pain at rest and during movement [4, 7]. Participants identified verbalisation, vital signs, and body language as instrumental in aiding pain assessment. In order to establish that the patient was in pain, the participants mostly ask the patients if they felt pain. The themes that emerged from the data presented in the study demonstrated how postoperative pain is assessed and managed by nurses in this hospital. The results of the study indicated that the participants mostly dwell on patients' verbalisation to assess pain. In our healthcare system, nurses play a vital role in assessing and managing postoperative pain; thus nursing care should follow an evidence-based paradigm that helps narrow the gaps between research and clinical practice. There are a wide range of approaches and tools available that can aid in the assessment and management of postoperative pain, but nurses are constrained in terms of skills and resources. It was also observed that the patient's facial expression was often one of the first indicators for the nurse that the patient is experiencing postoperative pain. Even though most participants rely on self-reports which is the most reliable method for identifying pain and widely accepted as the standard tool for identifying and measuring the pain experience, they may have challenges since it does not take into consideration individuals with cognitive deficits, who may not be able to verbally report their pain [29].

On the issue of utilisation of pain assessment scales, some of the nurses stated that they have never used any pain assessment tool and that the unit has no standard tool for assessing postoperative pain. Other participants reported the utilisation of the Numerical Rating Scale for pain assessment on very few occasions. The use of standardized scales has several advantages. They are reliable and objective and thus the accurate way to rate pain severity [30]; they take a short time to implement and the same scales can be used to assess the effectiveness of interventions [31]. Therefore with adequate training and with provision of resources, it is imperative that the use of standardized pain assessment scales could help in the proper assessment and management of postoperative pain in this setting. This study was done in the biggest teaching hospitals in Ghana and findings may not differ much among nurses across the country and in Africa as a whole where resources and skilled personnel are often lacking.

Postoperative pain management aimed to reduce or eradicate discomfort, prevent complications, facilitate the recovery, and achieve a pain-free state whenever possible [6]. Results from the study showed that nurses manage pain in such a way that pain was treated based on their own clinical experiences and not following any standard protocol. The majority of nurses reported that managing pain by using medication prescribed by the physician was what they always do for the patients, especially in the first 24 hours after surgery. Participants' reliance on medications to treat postoperative pain is in conformity with several studies [32] that indicate that the main goal of pain treatment is to relieve pain through the use of pharmacological interventions.

Nonpharmacological pain-relieving measures such as reassurance, diversional therapy, relaxation, and music therapy were employed by some nurses to manage postoperative pain. These interventions complement pharmacological pain treatment and nurses play a primary role in the incorporation of these methods in a patient's treatment plan [33]. However, the use of these measures was limited. This development confirmed a study by Brown and McCormack [34] that established that minimal consideration is given to nonpharmacological pain treatment in acute care of nursing practice. Although the use of particular music to divert the patient's attention away from pain and to promote a sense of relaxation and well-being has long been a popular approach, it was the least employed by participants. Music effectively reduces anxiety and improves mood for medical and surgical patients [35]. Perhaps, participants might not have been aware of the usefulness of nonpharmacological therapies in the management of postoperative pain. Also, these nonpharmacological interventions may have resource implications. Nurses are in short supply across all hospitals in Ghana, far below the recommended nurse to patient ratio and thus clinical activities may not allow nurses to spend time with the patient to enable them to use diversional therapies to manage pain.

## 5. Conclusions

Nurses' description of postoperative pain assessment and management indicated inaccuracy of postoperative pain assessment thus poor management of postoperative pain. The study provided several contributions to knowledge regarding the nature of assessment and management of postoperative pain at the Korle-Bu Teaching Hospital in Ghana. This includes the recognition of the surgical patients suffering due to the unsatisfactory pain management routines and the need to use more evidence-based approaches in postoperative pain assessment supported by validated pain assessment tools. Nurses were constrained to carry out their responsibilities. This issue must be seriously considered and discussed to ensure appropriate action can be taken for the benefit of delivering high quality nursing care for patients in pain. Another implication related to nursing practice is that this study would increase awareness of the health care professionals and the health institutions administration towards the establishment of team work to induce change, with a common purpose in upgrading the quality of pain assessment and management.

## Figures and Tables

**Table 1:** Examples of the themes and subthemes that emerged from the data analysis.

MEANING UNITS	CONDENSED MEANING UNITS	CODES	SUB-THEMES	THEMES
I think pain is what the person, the patient, says it is. If a patient mentions that he is in pain, it means that he is in pain, irrespective of what the health worker thinks. So post-operative pain is pain as a result of either a minor or major surgical procedure (N1)	(i) pain is what the patient says it is (ii) postoperative pain is as a result of surgery	(i) pain is subjective (ii) operational pain	(i) defining pain (ii) defining postoperative pain	Nurses' understanding of pain and postoperative pain
Pain is an unpleasant feeling that a patient expresses; it is an unpleasant sensory motor feeling that a patient or a person expresses and can only be expressed or be traced to what the patient verbalizes or says there is. (N3).	(i) unpleasant feeling that is expressed	(i) pain is subjective		

**Table 2:** Participants Characteristics.

Characteristics		Frequency
Age	20-30yrs	3
	31-40yrs	7
	Above 40yrs	2
Gender	Male	3
	Female	9
Qualification/Position	Staff Nurse	2
	Senior Staff Nurse	1
	Nursing Officer	4
	Senior Nursing Officer	4
	Principal Nursing Officer	1
Experience in nursing	1-2yrs	3
	3-5yrs	4
	6-10yrs	4
	Over 10yrs	1
Pre/post Training Programme	Critical Nursing	8
	Workshop on Pain	3
	No workshop on pain	1

## Data Availability

The data used to support the findings of this study are available from the corresponding author upon request.

## Additional Points

*Limitations of the Study*. This study adopts a qualitative approach and no attempt is made to generalise the findings to a wider context. However, detailed information is provided to inform the transferability of the study findings in a similar setting.
